# Exploring Medical Students’ Motivations to Apply for the Regional Quota Admission System: A Qualitative Study in Okinawa, Japan

**DOI:** 10.7759/cureus.94146

**Published:** 2025-10-08

**Authors:** Richi Kakazu, Yuiko Hiyajo, Miyu Yara, Hirotake Machida, Kiyoshi Kinjo, Ryuichi Ohta

**Affiliations:** 1 Family Medicine, Faculty of Medicine, University of Ryukyus, Okinawa, JPN; 2 Community Care, Unnan City Hospital, Unnan, JPN

**Keywords:** career choice, chikiwaku, health workforce, medical student admission, okinawa, physician distribution, qualitative research, regional quota, underserved populations

## Abstract

Background

The Faculty of Medicine at the University of the Ryukyus introduced a regional quota system to address the uneven distribution of physicians and specialties in remote islands and underserved areas of Okinawa Prefecture. Approximately 17 students are admitted annually under this system, which mandates graduates to work in designated regions or hospitals for a fixed period. Career choices are also constrained by limits on the number of students allowed into specific specialties. The motivations behind applying to the regional quota system remain varied and have not been sufficiently explored. This study aimed to clarify the reasons and motivations underlying students’ decisions to pursue the regional quota.

Methods

Semi-structured interviews were conducted with 12 regional quota students enrolled in their first to fourth years of study. Participants were selected through purposive sampling. Each interview lasted approximately 45-60 minutes and was conducted face-to-face using a structured interview guide. The discussions were recorded, transcribed verbatim, and coded. Thematic analysis was performed following established six-step procedures (familiarization, coding, generating themes, reviewing, defining, and interpretation). Among the 12 participants, three were male and nine were female. The Clinical Ethics Committee of Unnan City Hospital approved the study.

Results

Thematic analysis revealed that both practical and value-based factors influenced students’ decisions. Practical considerations included concerns about academic performance, anxiety over prolonged pre-admission periods, and financial benefits. Value-driven motivations included a strong desire to become a physician, commitment to serving their communities, and personal ties to remote islands. Many students also reported being influenced by family encouragement, financial support, and the pursuit of stability in life.

Conclusion

This study highlights the diverse motivations behind choosing the regional quota system. The findings suggest that clearer pre-enrollment information, structured mentorship, and flexible career support are needed to align students’ expectations with program obligations. These practical measures may strengthen students’ professional identity, increase satisfaction, and improve long-term retention in underserved areas.

## Introduction

The shortage and uneven distribution of physicians are critical healthcare challenges in Japan, particularly in remote islands and underserved areas where maintaining healthcare systems remains challenging [1,2]. Many medical universities, including the Faculty of Medicine at the University of the Ryukyus, have implemented regional quota systems to address these issues [3]. This system aims to alleviate physician shortages in remote and underserved areas of Okinawa Prefecture by training doctors through the regional quota program [4]. Under the regional quota system, approximately 17 students are admitted annually and are obligated to undergo training and work in designated regions or hospitals for nine years after graduation [4-6]. Additionally, restrictions on the number of physicians entering specific specialties are in place, which is expected to help mitigate disparities in specialty distribution [7-9].

Establishing the regional quota system is underpinned by a societal demand to reduce health disparities caused by the unequal distribution of medical resources [5,6]. While reports on the outcomes of regional quota students, such as their employment in designated areas and their distribution across specialties, exist, challenges remain in achieving the system's goals [7,8].

Current issues recruiting regional quota students include cases where applicants do not fully understand the system and the psychological burden of limited career options during the mandatory service period [9]. Motivations and reasons for choosing the regional quota system are diverse, with significant differences in individual aspirations and goals at the time of enrollment [10]. Understanding students’ motivations is crucial because they not only influence initial enrollment but also shape long-term commitment to serving in rural areas. Without clarity on these motivations, policymakers risk designing quota systems that fail to effectively address regional shortages. Accordingly, the objective of this study is to explicitly identify and analyze the key motivational factors influencing medical students’ decisions to apply for the regional quota system, using thematic analysis of semi-structured interviews.

## Materials and methods

Setting

This study aimed to explore the motivations and factors influencing the decision to choose the regional quota system at the Faculty of Medicine, University of the Ryukyus. The study was conducted with approval from the Clinical Ethics Committee of Unnan City Hospital (Approval ID: 20230039).

Study population

The participants included 12 regional quota students enrolled in their first to fourth year at the Faculty of Medicine, University of the Ryukyus. Participants were selected through purposive sampling. The purpose and significance of the research were explained to the participants, and informed consent for participation in the interviews was obtained. This study was performed from April 1 to September 30, 2024.

Interview content

To gain a deeper understanding of the reasons and motivations for choosing the regional quota system, as well as the system's impact on career choices and perspectives, the interview guide was structured around the topic: The Circumstances and Reasons for Choosing the Regional Quota System.

The concrete questions aimed to explore the participants' backgrounds and thought processes that led to their decisions. Questions addressed factors such as the influence of academic performance, financial incentives, family or community encouragement, and personal values, including a desire to serve remote or underserved areas. Participants were also asked about their understanding of the system at the time of application, including their knowledge of its obligations and potential limitations.

The interview guide included concrete questions designed to explore the participants’ backgrounds and thought processes leading to their decisions. For example, participants were asked: “How did your academic performance in high school or university influence your decision to apply?”; “Did financial incentives, such as scholarships or reduced tuition, affect your choice?”; and “What role did your family, teachers, or community play in encouraging your decision?” To explore personal motivations, participants were asked: “What values or goals, such as a desire to serve in remote or underserved areas, shaped your decision to apply?” In addition, the guide addressed participants’ prior understanding of the system, with questions such as: “What did you know about the obligations and commitments of the regional quota system when you applied?” and “Were you aware of any potential limitations or challenges associated with participation at the time of application?”

Analysis

Semi-structured interviews were conducted with participants, recorded, and transcribed verbatim to ensure the accuracy of the data. The transcripts were then analyzed using thematic analysis, a systematic method for identifying, organizing, and interpreting meaningful patterns within qualitative data [11].

The analysis followed six steps: familiarization with the data, coding the data, generating initial themes, reviewing and refining themes, defining and naming themes, and contextual interpretation [11]. Upon familiarizing myself with the data, I read the transcripts multiple times to gain a deep understanding of the content and context. Researchers reviewed the transcripts from first to fourth, gaining a deep understanding of the participants' contexts. This initial stage allowed the researchers to become familiar with the overall narrative and identify preliminary insights or patterns.

In coding the data, the data were segmented into meaningful units and assigned codes. These codes represented key ideas, phrases, or concepts related to participants’ motivations, experiences, and perspectives. Coding was done inductively to allow themes to emerge naturally from the data while remaining flexible to adapt to new insights. The first four researchers coded individually and then discussed their coding with the research team, including the RO, to refine it.

The codes were grouped into broader categories based on similarities or relationships in generating initial themes. This step involved clustering data into overarching themes that captured recurring patterns and differences in participants’ responses. In reviewing and refining the themes, they were examined against the raw data to ensure they accurately represented participants’ experiences and motivations. The research teams discussed the codes and developed initial themes. Overlapping or ambiguous themes were refined to enhance clarity and coherence, and sub-themes were created as needed to provide a more nuanced understanding. We deleted or merged the initial themes after identifying similarities among them during the team discussion.

We developed initial themes through continual discussion. By reviewing the initial themes, each theme was clearly defined and named to encapsulate its essence. This step aimed to articulate the relevance of the themes to the research questions and highlight how they contributed to understanding the motivations and experiences of regional quota students.

In the context of contextual interpretation, we developed final concepts and themes through continuous discussion, and as we progressed, we revisited the transcripts to refine them. The themes were interpreted concerning the broader context of the regional quota system, including its goals, challenges, and participants' lived experiences. Patterns such as shared motivations, unique challenges, or shifts in career perspectives were analyzed to provide meaningful insights. This iterative and rigorous process ensured that the analysis was comprehensive, credible, and reflective of the complexities within the data, offering actionable insights into the regional quota system's impact on students.

Reflexivity

This study was conducted collaboratively through interactions between the researchers and participants for the confirmability. The research team had diverse expertise and perspectives on rural community care and medical education. RK, HM, MY, and YH were medical students at the University of the Ryukyus. RK, MY, and YH were students from the regional quota. HM was a student not from the regional quota. Four of the researchers were engaged in the semi-structured interviews. RO is a family physician and public health professional who graduated with a master's degree in public health and family medicine and has experience conducting research on rural community healthcare. To minimize bias, each idea related to the conditions of motives among medical students from the regional quota was discussed by analyzing the research content of the individual data analyses. Alternative viewpoints were explored during the data interpretation stage.

## Results

A complex interplay of factors influencing their decision to enroll in the regional quota system was identified through a thematic analysis of the interviews conducted with 12 regional quota students at the University of the Ryukyus School of Medicine. The study participants consisted of 12 students: two were in their 4th year, six in their 3rd year, one in the 2nd year, and three in the 1st year. The gender distribution was three males and nine females. Regarding their background, nine students were from the regional quota, with origins on the main island of Okinawa. The findings revealed three overarching themes: (1) Conflict between Risks and Benefits, (2) Motivation Rooted in Community and Professional Aspirations, and (3) External Influence and Practical Considerations. Overall, enrolling in the regional quota system was a multifaceted decision, driven by personal aspirations, practical considerations, and external influences. While some participants prioritized the financial benefits and reduced academic pressure, others expressed profound motivation rooted in community service and professional identity development (Table 1).

**Table 1:** The concepts and themes of factors influencing the decision to enroll in the regional quota system Table 1 summarizes the concepts underpinning the three overarching themes. Rather than reiterating each item, we highlight how these patterns reveal tensions between external pressures and intrinsic motivation.

Theme	Concept
Conflict between Risks and Benefits of Choosing the Regional Quota	Conflict regarding admission type
	Anxiety about gap year
	Compromised choice due to concerns about academic performance
	Selection motivated by financial benefits
Motivation Rooted in Empathy for Supporting Regional Healthcare	Vague admiration for becoming a doctor
	Hope for a medical career inspired by personal experience
	Pre-admission experiences related to remote islands
	Strong desire to contribute to the local community
	Motivation driven by the aspiration to become a doctor
Support from Surroundings Seeking Stability in Life	Indifference toward practice location
	Strong encouragement from others
	Parental recommendation based on financial benefits

**Figure 1:**
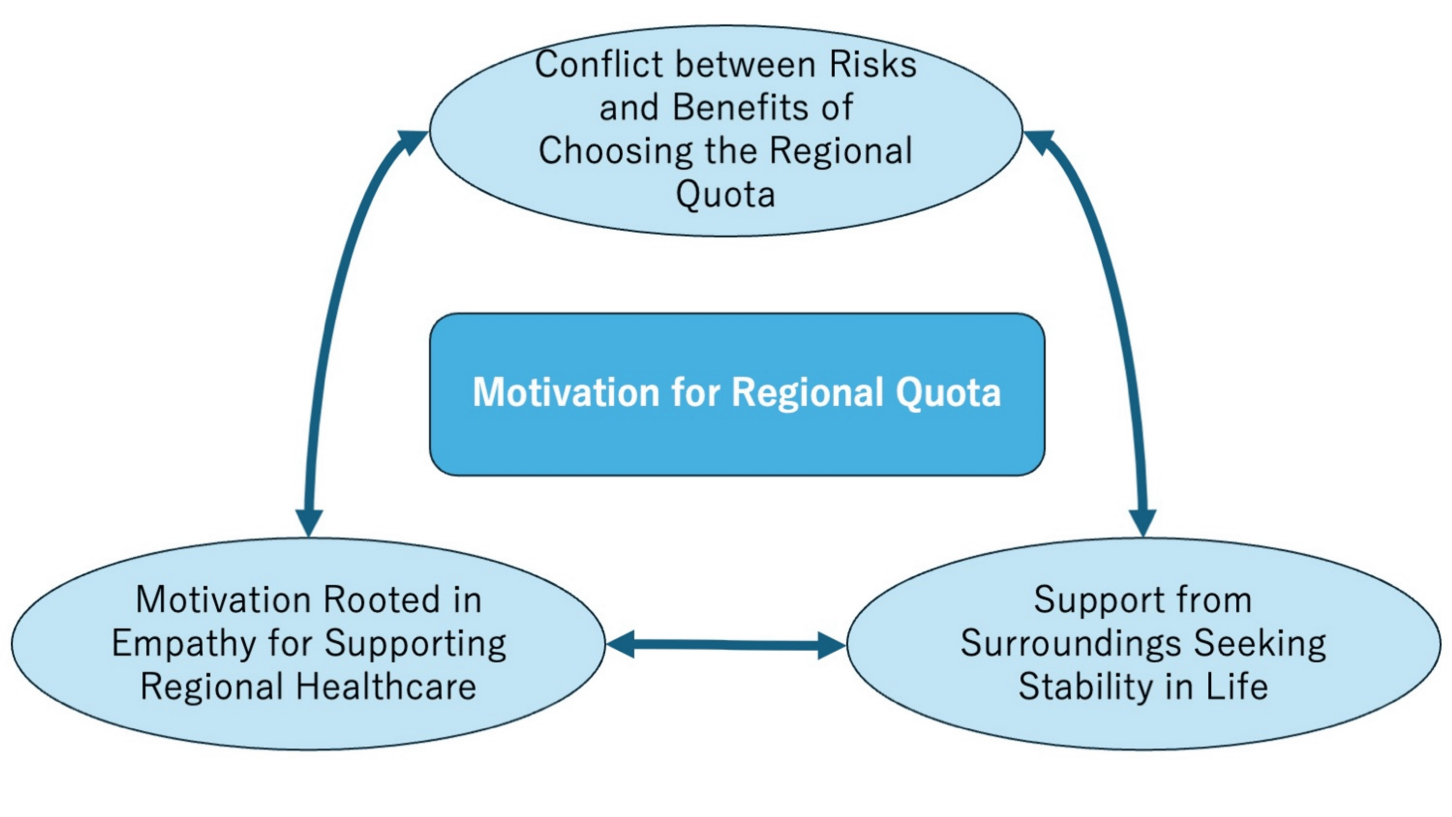
The concept figure of thematic analysis regarding factors influencing the decision to enroll in the regional quota system Figure 1 illustrates these interconnections, showing how practical and value-driven factors coexist in students’ decision-making. Image credit: Ryuichi Ohta

The number of participants was limited to 12, which is consistent with qualitative research aiming for depth rather than breadth. In thematic analysis, smaller samples allow for detailed exploration of participants’ narratives and enable the identification of nuanced themes across cases. The focus was therefore on achieving thematic saturation - the point at which no new concepts emerge - rather than statistical representativeness. This sample size is considered sufficient to provide theoretically meaningful insights into the motivations and decision-making processes of regional quota students in the Okinawan context. Figure 1 illustrates the conceptual framework of the thematic analysis.

Conflict between risks and benefits

The theme "Conflict between Risks and Benefits" captures the nuanced tensions students experienced in deciding to enroll in the regional quota system. Their choices were shaped by four major concepts: conflict regarding admission type, anxiety about gap year, compromised choice due to concerns about academic performance, and selection motivated by financial benefits. The interplay of these four concepts highlights the complexity of students’ decision-making processes when choosing the regional quota system. While the system provided a pathway to medical education, it also created significant tensions between immediate benefits and long-term sacrifices.

Conflict Regarding Admission Type

For many students, the decision to pursue the regional quota was influenced by a sense of compromise regarding the type of admission. They viewed the regional quota as a practical yet limiting alternative to general admission. Participant 10 explained, "I knew the regional quota came with obligations, but I had to weigh those against the uncertainties of competing in the general admission process. It felt like the safer route, even if it wasn't ideal." Some students described feeling powerless over their choice as external pressures or perceived barriers pushed them toward the regional quota. Another student noted, "It wasn’t my first choice, but I felt the regional quota was more accessible given my situation" (Participant 1). This sense of compromise highlights how the structure of admission pathways shapes students’ decisions.

Anxiety About Gap Year

The fear of experiencing another gap year significantly motivated many students. This anxiety often stemmed from the emotional and practical toll of delaying their medical education. A participant shared, "After a year of retaking exams, I didn’t want to go through that uncertainty again. The regional quota gave me a clear path forward" (Participant 1). Societal and familial expectations also influenced the pressure to avoid prolonged educational interruptions. One student remarked, "People around me said that failing again would reflect badly on me, and I didn’t want to risk disappointing my family. I chose the regional quota because it felt like the quickest way to move on" (Participant 9). These accounts illustrate how concerns about time and societal perceptions shaped students' decisions.

Compromised Choice Due to Concerns About Academic Performance

Concerns about academic performance were a recurring theme, particularly for students who perceived the regional quota as an opportunity to bypass the intense competition of the general admission process. A student said, "I wasn’t confident in my ability to get into medical school through the regular route, so the regional quota felt like a lifeline, even if it came with strings attached" (Participant 11). Another participant elaborated on this perspective: "I knew I wasn’t a top scorer, and I didn’t want to risk applying to general admission and failing again. The regional quota offered a way to fulfill my dream of becoming a doctor without the same pressure level" (Participant 6). These concerns reflect how the regional quota system attracts students who feel academically vulnerable, even as they grapple with its restrictions.

Selection Motivated by Financial Benefits

Financial incentives were a critical factor influencing students' decisions. For many, the financial relief provided by the quota system outweighed its limitations. One participant explained, "The tuition support was a huge factor for me. Without it, attending medical school would have been financially impossible for my family" (Participant 5). Parental influence often reinforced the appeal of financial benefits. A student shared, "My parents encouraged me to apply because they knew the financial support would ease our burden. They saw it as a stable option for my future" (Participant 2). Another participant reflected on the dual nature of this motivator: "It wasn’t just about the money-it was about finding a way to study medicine without adding more stress to my family’s finances" (Participant 10). These accounts reveal how economic considerations played a decisive role in students’ decisions, often blending with other factors like familial expectations.

Motivation rooted in community and professional aspirations

This theme reflects the emotional and values-driven motivations behind students' decisions to enroll in the regional quota system. The five concepts - Vague admiration for becoming a doctor, Hope for a medical career inspired by personal experience, Pre-admission experiences related to remote islands, Strong desire to contribute to the local community, and Motivation driven by the aspiration to become a doctor - collectively highlight the multifaceted nature of their ambitions. The interplay of these five concepts illustrates the depth and diversity of motivations among students who chose the regional quota system. Whether inspired by personal experiences, community ties, or a lifelong admiration for medicine, these students viewed their enrollment as an opportunity to make a meaningful impact. Recognizing these varied motivations is essential for designing supportive systems that nurture their commitment to underserved communities while helping them achieve their personal and professional goals.

Vague Admiration for Becoming a Doctor

For some students, pursuing a career in medicine was rooted in a general admiration for the profession, often formed during childhood. A participant described this as a nebulous but persistent dream: "I didn’t know exactly what being a doctor entailed, but I always thought it was an incredible and noble profession" (Participant 6). Another shared, "As a high school student, I admired doctors without fully understanding their role. This vague idea of wanting to help people and do something meaningful guided me" (Participant 2). While initially abstract, this admiration often became more focused over time through personal and educational experiences.

Hope for a Medical Career Inspired by Personal Experience

Many students described how personal experiences with healthcare, either as patients or through family members, had solidified their desire to pursue a career in medicine. One participant shared, "When my friend was hospitalized, I saw how much the doctors did to save their life. That experience stuck with me and made me want to be someone who could do the same for others" (Participant 9). Another reflected on their family’s struggles with healthcare access: "My grandmother had chronic illnesses, and seeing how kind and dedicated the doctors were made me realize how much of a difference they made in our lives" (Participant 12). These profoundly personal encounters often were pivotal moments that transformed admiration into action.

Pre-Admission Experiences Related to Remote Islands

Students who grew up on remote islands or in underserved areas often cited their pre-admission experiences as central to their decision. A participant explained, "Growing up on a small island, I saw firsthand how hard it was to access healthcare. It made me realize the importance of having doctors in these areas" (Participant 3). Another added, "I volunteered at a local health center on my island during high school. That experience showed me how much people depended on our few medical professionals and how fulfilling it could be to serve in such a role" (Participant 12). These experiences cultivated a strong sense of awareness about the challenges faced by rural and underserved communities, driving their commitment to returning as healthcare providers.

Strong Desire to Contribute to the Local Community

A profound duty to give back to their communities was a recurring theme. Many students felt a moral obligation to improve healthcare in underserved regions where they had personal or familial ties. One participant reflected, "I always felt grateful for the community that supported me growing up. Becoming a doctor and returning to help them felt the best way to repay that kindness" (Participant 6). Another shared, "I want to ensure that future generations in my hometown don’t struggle to find medical care like my family did. It’s not just a career for me" (Participant 4). This commitment was often profoundly personal, driven by students’ intimate knowledge of the disparities their communities faced.

Motivation Driven by the Aspiration to Become a Doctor

For some students, the decision to enter the regional quota system was not just about fulfilling a specific obligation or addressing a healthcare gap but about realizing their aspiration to become a doctor. One student shared, "I’ve always wanted to be a doctor, and the regional quota system allowed me to achieve that goal. It didn’t matter where I worked. I just wanted to make it happen" (Participant 10). Another echoed this sentiment, saying, "Medicine was always my dream, and the regional quota was the path that allowed me to follow it. It aligned with my values and gave me a clear direction" (Participant 5). This overarching aspiration was often intertwined with other motivations but stood out as a driving force for many participants.

External influence and practical considerations

The theme "External Influence and Practical Considerations" delves into how financial stability, family expectations, and societal pressures shaped students' decisions to enroll in the regional quota system. These influences were often intertwined, highlighting the practical realities that students faced in pursuing their aspirations within the constraints of their personal and social circumstances. The decision to enroll in the regional quota system was often rooted in a complex interplay of external influences and pragmatic considerations. For many students, financial incentives were the foundation upon which their decisions were built, alleviating economic barriers that would have otherwise hindered their ability to pursue medical education. Family expectations provided additional motivation, with parents often advocating for this pathway to ensure financial stability and future security. Community recommendations further reinforced these decisions, especially for students uncertain about their preferences or lacking strong opinions about their career direction.

Financial Incentives

Financial support provided by the regional quota system stood out as one of the most decisive factors influencing students’ choices. Many participants described how this support not only made medical education accessible but also reduced the financial strain on their families. The system’s tuition subsidies offered a lifeline to students from economically constrained backgrounds, enabling them to pursue their dreams without the burden of crippling debt. One participant described the critical nature of this support, stating, "The tuition support was critical since my family couldn't afford private medical school" (Participant 5). The financial relief outweighed the program’s restrictions for these students, making the regional quota system an attractive and pragmatic choice.

Family Expectations

Family influence played a substantial role in shaping students’ decisions, often as a guiding force behind their enrollment in the regional quota system. Parents frequently encouraged their children to choose this pathway, seeing it as a financially secure and stable option for their future. This encouragement was particularly evident in families where financial limitations created pressure to seek cost-effective educational opportunities. One participant shared how their parents’ perspective shaped their decision: "My parents encouraged me to apply because they knew it would be a financially stable option" (Participant 12). Students were often motivated to fulfill their parents' expectations and contribute to their family’s stability and pride.

Community Recommendations

Recommendations and encouragement from community figures, such as high school teachers or local mentors, also influenced students' decisions. For some, these external voices provided reassurance and clarity, especially when students felt uncertain about their future career path or lacked strong preferences for practice locations. One participant recounted, "I didn't have a strong opinion about where I would work, so when my high school teacher recommended the regional quota, it felt like the right decision" (Participant 1). Such guidance was often pivotal, steering students toward the regional quota system as a practical and respectable choice.

## Discussion

This study explored the motivations and decision-making processes of medical students who enrolled in the regional quota system at the Faculty of Medicine, University of the Ryukyus. The analysis revealed three overarching themes: (1) conflict between risks and benefits, (2) motivation rooted in community and professional aspirations, and (3) external influence and practical considerations. The regional quota system provided students with a pathway to medical education, often through reduced competition, financial support, and quicker enrollment. However, this came with long-term obligations such as restricted specialty choices and mandatory service in specific areas. Many students perceived this as a “compromised choice” when opting for the regional quota system [7]. Some saw it as a lifeline amid academic or financial difficulties, while others expressed concern about external influence and practical considerations. These findings align with previous studies indicating that medical students’ decision-making is strongly shaped by external pressures and the desire for stability, particularly in resource-constrained settings [12-14].

A strong commitment to contributing to underserved communities also emerged as a powerful motivator, particularly among students who grew up on remote islands or had witnessed healthcare disparities. This aligns with the program’s stated mission of addressing healthcare inequities in Okinawa [10,15,16]. Yet the degree of intrinsic motivation varied [17]. Some students articulated a clear vision of their role in community health, while others entered with more abstract aspirations that developed during medical school. Supporting students in refining and sustaining their professional identities may enhance commitment to the program’s goals [18]. Mentorship programs, community engagement activities, and structured opportunities for reflection have been suggested to strengthen motivation for service in underserved areas [19,20].

Practical considerations such as financial incentives, family expectations, and community recommendations further influenced students’ choices. Financial support was particularly crucial for students from economically constrained backgrounds, reinforcing the regional quota system’s role in widening access to medical education [21,22]. However, relying solely on financial incentives risks overshadowing the broader purpose of cultivating intrinsic motivation. Family expectations and recommendations from community mentors also played an important role [23], but these external pressures could contribute to dissatisfaction or disengagement if intrinsic motivation were lacking [24].

These findings can also be situated within established theoretical frameworks. According to Self-Determination Theory (SDT), motivation exists along a continuum from externally regulated (e.g., financial incentives, family encouragement) to intrinsically regulated (e.g., aspirations to serve communities, professional identity) forms. Prior studies have shown that fostering autonomy, competence, and relatedness in educational settings promotes the internalization of external motives into more sustainable intrinsic ones [25]. Additionally, Social Cognitive Career Theory (SCCT) emphasizes the importance of self-efficacy, outcome expectations, and contextual supports in shaping career decisions [26]. In our findings, concerns about academic performance reflect low self-efficacy, whereas pre-admission experiences in remote areas and admiration for physicians represent positive outcome expectations. Family and community encouragement act as contextual supports that help translate these beliefs into concrete goals, consistent with previous studies on rural medical workforce pathways [27-29]. Integrating these theoretical perspectives highlights how both internal dispositions and external environments shape students’ motivations.

Importantly, motivation is not static but evolves. While some students initially rely on external drivers such as financial support or reduced competition, sustained exposure to community-based training and mentorship may cultivate more substantial intrinsic commitments to rural service. Conversely, if intrinsic motivation is not reinforced, there is a risk of disengagement from quota obligations, which can potentially affect retention and specialty distribution in underserved areas. Longitudinal and multi-institutional studies are therefore needed to examine how motivational trajectories shift across medical training and mandatory service periods, and how these trajectories influence actual career retention in rural practice [30].

In addition to mentorship and community engagement, other teaching approaches may also enhance motivation and professional identity formation. Special study modules (SSMs) and team-based learning (TBL) have been reported to promote greater proficiency in specific domains, such as imaging techniques, while also fostering collaboration and integrative learning. A recent comprehensive evaluation demonstrated that SSMs were effective across key educational domains, particularly in facilitating the integration of knowledge and promoting teamwork among participants [31]. Incorporating such methods into quota student curricula could provide structured opportunities to link theoretical knowledge with practical skills, thereby strengthening both competence and commitment to rural practice.

This study has several limitations. First, the sample was small and relatively homogeneous (n = 12), and it was drawn from a single institution in Okinawa. However, thematic saturation was reached; this composition, including a predominance of female participants (9/12), may have constrained the range of perspectives and limited the exploration of gendered differences, thereby affecting transferability to other settings. Second, the study relied on self-reported, cross-sectional interview data, which may be subject to social-desirability and recall biases, and there was no longitudinal follow-up. Third, we did not triangulate data sources (e.g., faculty or administrative perspectives, documentary materials, or quantitative surveys), which may have reduced the credibility and completeness of the findings. Fourth, several interviewers were student peers; despite safeguards (independent coding by multiple researchers, iterative team discussions, anonymization, and reflexive practice), peer familiarity may have influenced responses. Finally, our analysis prioritized thematic synthesis over subgroup comparisons; therefore, we did not formally investigate how demographics (e.g., gender, year of study) influenced the themes. Future work should use larger, multi-institutional, and more gender-balanced samples, incorporate methodological triangulation and longitudinal or mixed-methods designs, and explicitly test demographic patterning to enhance credibility and transferability. In addition, while motivations were analyzed in terms of themes, we did not examine measurable learning-style dimensions. Prior studies suggest that students’ individual learning preferences - visual, auditory, or kinesthetic - play a significant role in their engagement, study duration, and academic success [32]. Our interpretive approach may therefore have overlooked how background, perception, and institutional context interact with these learning styles to shape motivation and professional identity. Future work should integrate both interpretive analyses and structured assessments of learning preferences to provide a more holistic understanding.

## Conclusions

The findings of this study shed light on the complex interplay of motivations and factors influencing students’ decisions to enroll in the regional quota system. While the program provides essential opportunities for medical education and aims to address healthcare disparities, it also presents significant challenges that require careful consideration. In practical terms, our results suggest that policymakers and educators should provide clearer pre-enrollment information, strengthen mentorship and career counseling, and offer flexible pathways that balance service obligations with students’ career aspirations. Such measures could enhance students’ satisfaction, professional identity formation, and long-term retention in underserved areas. By tailoring support systems to students’ diverse motivations and addressing their concerns about career restrictions, the regional quota system can be further refined to improve healthcare access in underserved regions.
